# Metabolomic Differences between the Skin and Blood Sera of Atopic Dermatitis and Psoriasis

**DOI:** 10.3390/ijms232113001

**Published:** 2022-10-27

**Authors:** Liis Ilves, Aigar Ottas, Bret Kaldvee, Kristi Abram, Ursel Soomets, Mihkel Zilmer, Viljar Jaks, Külli Kingo

**Affiliations:** 1 Department of Dermatology and Venereology, University of Tartu, 50417 Tartu, Estonia; 2 Dermatology Clinic, Tartu University Hospital, 50417 Tartu, Estonia; 3 Department of Biochemistry, Institute of Biomedicine and Translational Medicine, University of Tartu, 50411 Tartu, Estonia; 4 Centre of Excellence for Genomics and Translational Medicine, University of Tartu, 50411 Tartu, Estonia; 5 Department of Cell Biology, Institute of Molecular and Cell Biology, University of Tartu, 51010 Tartu, Estonia

**Keywords:** atopic dermatitis, psoriasis, metabolomics, sphingolipids and inflammation

## Abstract

Atopic dermatitis (AD) and psoriasis (PS) are common chronic inflammatory dermatoses. Although the differences at the intercellular and intracellular signaling level between AD and PS are well described, the resulting differences at the metabolism level have not yet been systematically analyzed. We compared the metabolomic profiles of the lesional skin, non-lesional skin and blood sera of AD and PS. Skin biopsies from 15 patients with AD, 20 patients with PS and 17 controls were collected, and 25 patients with AD, 55 patients with PS and 63 controls were recruited for the blood serum analysis. Serum and skin samples were analyzed using a targeted approach to find the concentrations of 188 metabolites and their ratios. A total of 19 metabolites differed in the comparison of lesional skins, one metabolite in non-lesional skins and 5 metabolites in blood sera. Although we found several metabolomic similarities between PS and AD, clear differences were outlined. Sphingomyelins were elevated in lesional skin of AD, implying a deficient barrier function. Increased levels of phosphatidylcholines, carnitines and asymmetric dimethylarginine in PS lesional skin and carnitines amino acids in the PS serum pointed to elevated cell proliferation. The comparison of the metabolomic profiles of AD and PS skin and sera outlined distinct patterns that were well correlated with the differences in the pathogenetic mechanisms of these two chronic inflammatory dermatoses.

## 1. Introduction

Atopic dermatitis (AD) and psoriasis (PS) are common chronic inflammatory dermatoses. PS is an immune-mediated disease that affects approximately 1.5–3% of the population [1]. The pathogenesis of PS involves Th1-, Th17- and Th22-lymphocyte driven inflammatory cascade, keratinocyte hyperproliferation and altered differentiation, as well as angiogenesis and vasodilatation [2,3,4]. Genetic and environmental factors also play a role in the development and persistence of the disease [5]. The prevalence of AD is 1–7% in adults, up to 20% in children and it has increased during past few decades [6,7]. AD is characterized by an impaired barrier function and a predominantly dysregulated Th2-mediated inflammation, although other immune pathways are included as well. The disease has a genetic predisposition and is worsened by numerous environmental factors such as reduced humidity, pollutants and tobacco smoke [8,9,10,11].

The differences at the intercellular and intracellular signaling level between AD and PS are well described; however, the resulting differences at the metabolism level have not been systematically analyzed yet. Both AD and PS are multifactorial diseases and metabolomic studies can help in better understanding their pathogenesis, which could lead to finding novel treatment opportunities in the future. To date, different approaches have been used to identify biomarkers characteristic of AD and PS.

In psoriatic lesional (PS-L) skin, there have been found elevated arachidonic acid, 12-hydroxyeicosatetraenoic acid (12-HETE), leukotriene B4, PGE2, PGF2-alpha and 13-hydroxyoctadecadienoic acid, 8, 12- and 15-hydroxyeicosatetraenoic acid, choline and taurine concentrations and decreased levels of *myo*-inositol and glucose [12,13,14,15,16]. According to Sorokin et al., there are higher glycerol and acylcarnitine levels and lower glutathione and gamma-glutamyl levels in PS plasma compared with the healthy controls [15]. Armstrong et al. found higher amounts of alpha ketoglutaric acid and lower amounts of asparagine and glutamine in PS patients’ blood serum (both with and without PsA) compared to controls. Elevated levels of alpha ketoglutaric acid and decreased levels of lignoceric acid were found in only cutaneous PS patients’ serum compared to PS patients with PsA. Higher concentrations of glucuronic acid were found in the serum of PS patients with PsA compared to controls [17]. In the comparison of the blood plasma of PS patients and healthy controls, differences among 37 amino acids (AA) and 40 carnitines were found. Negative correlations with PASI score were found in the concentrations of glutamine, asparagine and hexadecanoylcarnitine (C16) [18]. The lipidomic analysis of plasma samples collected from PS patients and healthy controls showed elevated levels of lysophosphatidic acid, lysophosphatidylcholine and phosphatidic acid and decreased levels of phosphatidylinositol and phosphatidylcholine in PS patients [19].

Less studies have been conducted on the metabolomic profile of AD. Berdyeshev et al. have found a higher proportion of short-chain ceramides, sphingomyelins and 14:0–22:0 lysophosphatidylcholines (LPC) and a decreased number of long-chain sphingolipids and LPC-s in AD skin compared to C [20]. In the serum of children with AD, decreased levels of glycine and taurine conjugated bile acids and increased levels of cholic acid, chenodeoxycholic acid and some unsaturated fatty acids were found compared to the controls [21]. In the urine of AD children, the concentrations of creatinine, creatine, citrate, formate, 2-hydroxybutyrate, dimethylglycine and lactate were increased and the concentrations of betaine, glycine and alanine were decreased [22].

Research related to metabolomic studies has been concentrated on the differences between lesional and non-lesional skin or that of blood sera of patients and healthy controls; nevertheless, the direct comparisons of the metabolomic profiles of PS and AD are virtually lacking. Here we elucidated for the first time the differences at the metabolism level in the PS and AD lesions and blood sera by head-to-head comparison. We detected a few similarities; however, we also unveiled remarkable differences (e.g., in the levels of phosphatidylcholines (PC) and sphingolipids) that corroborate the different pathogenetic mechanisms underlying these two widespread cutaneous pathologies.

## 2. Results

The concentration of each metabolite was normalized to values between 0 and 1 so that the metabolite levels of skin and serum could be compared. R package version 3.5.1 was used for data analyzation [23]. Non parametric Kruskal–Wallis rank sum test and the Mann–Whitney–Wilcoxon rank-sum test were used for statistical analysis. Benjamini–Hochberg (false discovery rate; FDR 5%) corrected *p* value < 0.05 was considered statistically significant.

**Figure 1:**
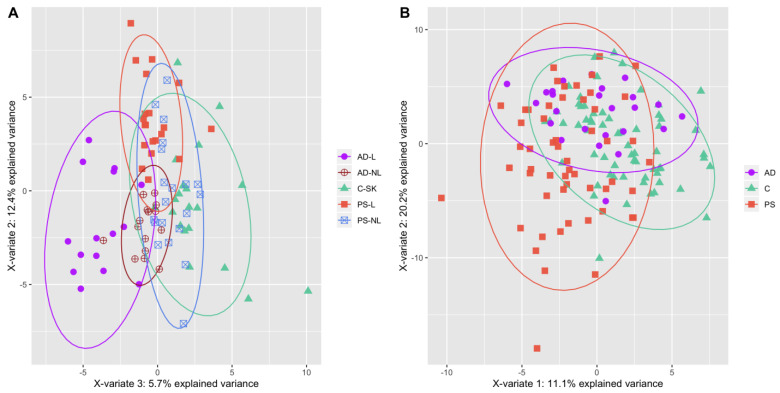
(**A**) PLS-DA plot of the samples of AD (lesional skin—filled violet circles, non-lesional skin—empty violet circles), PS (lesional skin—filled red squares, non-lesional skin—empty blue squares) and C (filled green triangles) skin. (**B**) PLS-DA plot of the samples of AD (violet circles), PS (red squares) and C (green triangles) sera. Data is based on metabolites that changed statistically significantly from Kruskal–Wallis test.

In this study, we compared the metabolome of skin and sera of AD and PS patients (Figure 1). We found 19 metabolites and their ratios that differed statistically significantly between atopic dermatitis lesional (AD-L) and psoriatic lesional (PS-L) skin (Table 1). The median concentrations of sphingomyelins (SM) [SM.C26.0 (*p* = 0.0028), SM.C26.1 (*p* = 0.0035), hydroxysphingomyeline C22.1 (SM.OH.C22.1; *p* = 0.0095), SM.C18.1 (*p* = 0.0095), SM.C24.1 (*p* = 0.0095), SM.C24.0 (*p* = 0.0294), hydroxysphingomyeline C22.2 (SM.OH.C22.2; *p* = 0.0328), SM.C16.1 (*p* = 0.044) and SM.C16.0 (*p* = 0.044)] and the ratio of ornithine to arginine (Orn…Arg; *p* = 0.0285) were statistically significantly higher in AD-L skin when compared to PS-L skin. The median concentrations of asymmetric dimethylarginine (ADMA; *p* = 0.0043), acetylcarnitine (C2; *p* = 0.0086) and octadecadienylcarnitine (C18.2; *p* = 0.0318), and a group of glycerophospholipids [PC acyl-alkyl C38:1 (PC.ae.C38.1; *p* = 0.0095), PC acyl-alkyl C36:0 (PC.ae.C36.0; *p* = 0.0184), lysophosphatidylcholine acyl C16:1 (lysoPC.a.C16.1; *p* = 0.0274), PC acyl-alkyl C38:0 (PC.ae.C38.0; *p* = 0.0365), PC acyl-alkyl C38:2 (PC.ae.C38.2; *p* = 0.044) and PC acyl-alkyl C36:1 (PC.ae.C36.1; *p* = 0.044)] were increased in PS-L skin when compared to AD-L skin.

When comparing atopic dermatitis non-lesional (AD-NL) and psoriatic non-lesional (PS-NL) skin, we found just one metabolite, acetylcarnitine (C2; *p* = 0.0481), that was more abundant in PS-NL samples, underpinning the similarity of NL skin samples as opposed to the lesional ones (AD-NL median value was 0.0985, PS-NL median value was 0.1455, 1.5-fold change).

In the comparison of AD and PS sera, we found five metabolites that were present at significantly different concentrations (Table 2). All the metabolite concentrations were elevated in PS sera, namely citrulline (Cit; *p* = 0.0392), glutamate (Glu; *p* = 0.0392), proline (Pro; *p* = 0.0392), carnitine (C0; *p* = 0.0184) and octadecenoylcarnitine (C18.1; *p* = 0.0392).

## 3. Discussion

As expected, the lesions of PS and AD were characterized by distinct metabolomic patterns. A group of SM-s was elevated in AD-L skin and a group of PC-s had elevated concentrations in PS-L skin. Among SM-s, all except SM.C16.1 were also elevated in AD-L skin when compared to C skin [24]. Notably, there were no differences in the concentrations of SM-s between PS-L, PS-NL and C skin [25].

Lipids in stratum corneum have water-retaining properties and provide protection from environmental factors. Ceramides are the essential constituents of stratum corneum lipids and consist of a fatty acid residue and a sphingoid base [26,27,28]. Notably, the number of ceramides is reduced in the skin of AD patients [29,30]. Ceramides are produced either by degrading glucosylceramides (GCer) and SM by glucosylceramidase and sphingomyelinase or formed from serine and palmitic acid by serine palmitoyltransferase and ceramide synthase. Conversely, ceramides can be metabolized to SM via sphingomyelin synthase [26,27,31,32,33]. Hara et al. have found an increased expression of SM deacylase in AD patients’ skin, which competes with sphingomyelinase and glucosylceramidase for SM and GCer [27]. Imokawa et al. also found 5-fold enhanced activity of SM-GCer deacylase (also referred to as SM deacylase) in AD-L skin [34]. Additionally, reduced sphingomyelinase activity has been reported in AD skin [35] and all these changes are expected to result in decreased levels of ceramides in AD skin. Thus, the increased concentrations of SM in atopic dermatitis skin potentially reflect the reduction in ceramide synthesis when compared to psoriatic lesions.

The potential explanation for such changes is the prevalence of Th2-mediated inflammation in the AD lesional skin. Berdyshev et al. demonstrated the role of type 2 immune response on the lipid composition of AD skin. Besides showing the decrease in very long-chain fatty acids in several lipid classes, they also noted the increase in SM-s in AD skin, the skin of IL-13 transgenic mice and IL-4/IL-13 treated differentiated keratinocytes [20]. Another report demonstrated that Th2 cytokines provoked a decrease in stratum corneum ceramide levels and also downregulated the expression of serine-palmitoyl transferase-2, acid sphingomyelinase and β-glucocerebrosidase (glucosylceramidase) [20,36]. At the same time, Th1 cytokines induced mild elevation of ceramides and either upregulated or did not alter the activity of mentioned enzymes.

Conversely, in PS-L skin, a subset of PC-s (PC.ae.C36.0, PC.ae.C38.0 and PC.ae.C38.1) were elevated when compared to C and AD-L skin. Additionally, C2 and lysoPC.a.C16.1 were elevated in PS-L skin when compared to PS-NL skin. PC-s are major constituents of cellular membranes. As epidermal hyperproliferation is a characteristic of PS, the increased amount of PC-s in psoriatic plaques is consistent with the increased cell division rate. Choline, a constituent of PC, has previously been found elevated in PS-L skin and its levels were positively correlated with the severity of PS [16,25,37].

Besides being structural components of the cells, PC-s also participate in intra and intercellular signaling as well as act as important substrates for the biosynthesis of other signaling molecules such as diacylglycerol and arachidonic acid [32,38,39]. The latter can further be metabolized to leukotrienes and prostaglandins and leukotriene B4, PGE2 and PGF2-alpha, as well as arachidonic acid, were found at elevated concentrations in PS-L skin [12,13,14]. In addition, LysoPC-s are likely to participate in PS inflammatory response, as these possess pro-inflammatory properties and amplify the function of the cells of innate and adaptive immunity [40].

When studying the amino acid metabolism, we found that Orn…Arg was significantly lower in PS-L skin when compared to AD-L skin. We have previously found that this was also decreased in the comparison of PS-L and C skin [25]. ADMA levels were also higher in PS-L skin compared to AD-L skin. ADMA blocks the synthesis of Cit from Arg via inhibiting nitric oxide synthase (NOS) and in line with this the median concentrations of Arg have been previously found elevated in both PS-L and AD-L skin when compared to the C skin. Furthermore, Bilgic et al. found elevated ADMA levels in PS patients’ blood compared to controls [41]. Elevated serum levels of ADMA have been associated with an increased risk of atherosclerosis and cardiovascular disease, diseases that are also more prevalent in PS patients [42,43,44].

Two carnitines (C2 and C18.2) were elevated in PS-L skin when compared to AD-L skin. Additionally, C2 levels were also increased in PS-NL skin when compared to AD-NL skin. Of note, C2 was previously found elevated in AD-L skin compared to AD-NL skin and in PS-L skin compared to PS-NL skin. C18.2 had higher concentrations in PS-L skin while compared with PS-NL and C skin [24,25].

Carnitine takes part in fatty acid metabolism by transporting fatty acids into the mitochondria. This process is essential for utilization of medium- and long-chain fatty acids and involves esterifying L-carnitine into various acylcarnitine derivatives such as C18.2. The hyperproliferation of keratinocytes needs additional energy which could be provided by fatty acid β-oxidation that takes place in mitochondria. Additionally, C2 is a marker of free CoA and thus acts as a marker of the cell’s energetic homeostasis [45].

In the serum of PS patients, the levels of C0 and C18.1 were elevated compared to AD serum. Ottas et al. have previously found decreased ratio of acetylcarnitine to free carnitine (C2…C0) in PS and AD sera, potentially indicating an altered lipid metabolism in these chronic dermatoses [46,47].

Hyperproliferation also increases the need for protein synthesis and Glu has a significant role in stabilizing protein structure. Glu is synthesized from α-ketoglutarate and from other amino acids (AA-s) such as glutamine, Pro, Arg and histidine [48]. We found elevated levels of Glu in PS blood compared to C and also PS-L skin compared to PS-NL and C skin, as well as in AD-L skin compared to AD-NL and C skin. Dutkiewicz et al. found a positive correlation between the levels of Glu in PS skin and the severity of PS [37]. Elevated Glu levels have been found in the sera of patients suffering from inflammatory diseases or frequent PS comorbidities such as non-alcoholic fatty liver disease, diabetes, cardiometabolic disease and atherosclerosis [49,50,51].

Pro was also elevated in PS blood compared to AD blood. Pro can be metabolized to Glu and vice versa. Additionally, Pro can be synthesized from Orn and be metabolized to Orn, being subsequently converted into putrescine or Cit [52]. This is in concordance with Kang et al., who also found elevated concentrations of Pro in PS blood plasma compared to controls [53]. Kamleh et al. found changes in three metabolic pathways (Arg and Pro; glycine, serine and threonine; alanine, aspartate and Glu) when comparing the plasma from patients with mild and severe PS from healthy controls and from patients with severe PS after treatment with Etanercept. There was an increase of 90% in Cit levels in severe PS compared to C and its levels also correlated positively with PASI scores [54]. The elevated concentration of Cit is consistent with our findings as its level was elevated in PS blood serum when compared with AD.

On the other hand, Bilgic et al. found that the serum of PS contained significantly higher amounts of ADMA as well as homocysteine and had lower values of Cit and L-Arg/ADMA [41]. We and other authors have found alterations in urea cycle intermediates previously in PS and also in AD patients’ blood and skin [24,25,37,41,46,55]. Recently, Lou et al. indicated that interleukin-17, a major driver of inflammation in PS induces alterations in urea cycle metabolite balance [56].

In conclusion, the comparison of the metabolomic profiles of AD and PS skin and sera outlined distinct patterns that were well correlated with the differences in the pathogenetic mechanisms of these two chronic inflammatory dermatoses. These differences encompassed several lipid classes, carnitines and alterations in the levels and ratios of urea cycle intermediates. In AD skin, altered lipid composition (increased SM-s) implied deficient barrier function. In PS skin, elevated PC-s denoted increased cell proliferation and acted as a potential source for inflammatory mediators. Increased ADMA and decreased ratio of Orn to Arg pointed to alterations in the urea cycle that in turn is possibly a hallmark of increased nucleic acid metabolism due to the cell proliferation. Elevated carnitine levels represented the alterations in fatty acid β-oxidation, which was also seen in the comparison of the blood sera of PS and AD patients. Elevated concentrations of AA-s and changes in the concentrations of urea cycle intermediates in PS blood sera indicated the increased need for protein synthesis in PS. As the previous findings at the cytokine levels have led to the development of biological therapy, the metabolomic studies might add valuable knowledge to the derivation of novel topical treatments and may also contribute to the better understanding of the mechanism of non-biological disease modulating medications.

## 4. Materials and Methods

### 4.1. Volunteer Recruitment

Adult patients with PS and AD and healthy controls were recruited from the Tartu University Hospital, Dermatology Clinic and Traumatology and Orthopaedics Clinic between 2013 and 2015 (Table 3). Inclusion criterium was the diagnosis of PS or AD. Skin biopsies from 15 patients with AD (4 men, 11 women, ages 20–50), 20 patients with PS (13 men, 7 women, ages 20–75, median PASI 7.7) and 17 controls (10 men, 7 women, ages 23–75) were collected. The AD and PS biopsies were collected during the acute phase of the disease. For the blood serum analysis, 25 patients with AD (6 men, 19 women, ages 19–54), 55 patients with PS (37 men, 18 women, ages 20–75, median PASI 9) and 63 controls (37 men, 26 women, ages 23–75) were recruited. All participants were Caucasians of Eastern European descent. Exclusion criteria for the patients and controls were any other concomitant skin disease, hypertension, diabetes, gout and regular medication intake.

### 4.2. Skin Biopsies

Three millimeter punch biopsies were taken from the lesional skin and adjacent (1–2 cm from lesions) non-lesional skin from the upper arm and torso of patients with AD and PS, as well as from similar locations of non-sun-exposed skin of controls. Subsequently, skin samples were frozen immediately in liquid nitrogen and stored at −80 °C until needed. The period for collecting the samples was 3 years, after which metabolites were extracted and samples were lyophilized and stored at −80 °C until analysis, as described previously [57]. Before the measurements, the skin samples were weighed, and a mix of 12 mL/g(sample) methanol and chloroform and 6 mL/g(sample) water was added. Steel balls (12 mm diameter) were added to the tube and milled using BulletBlender (NextAdvance). The sample was incubated for 1 h on ice, the supernatant was transferred to a clean tube and centrifuged at 16,000× *g* and 4 °C for 15 min. The methanol/water and chloroform phases were pipetted to separate tubes and lyophilized.

### 4.3. Blood Samples

Fasting blood samples were collected into 5 mL Vacutainer (REF 367614) tubes with micronized silica particles. The samples were centrifuged at 1300× *g* for 20 min and were left to clot for one hour at room temperature. The supernatant was aliquoted into 300 μL fractions and stored at −80 °C.

### 4.4. Metabolomic Analysis

For the targeted analysis of 188 metabolites and their ratios, the AbsoluteIDQ p180 kit (Biocrates Life Sciences AG, Innsbruck, Austria) was used according to the manufacturer’s instructions. For further analysis, Agilent Zorbax Eclipse XDB C18, 3.0 × 100 mm, 3.5 μm with Pre Column SecurityGuard, Phenomenex, C18, 4 × 3 mm was used on a 1260 series HPLC (Agilent, Santa Clara, CA, USA) in tandem with a QTRAP 4500 (ABSciex, Framingham, MA, USA) mass spectrometer.

Lyophilized skin samples were thawed on ice, dissolved in 85% methanol/15% water according to their previous weight (15–25 μL added solvent) and pipetted to the filter plate along with 10 μL internal standards. The samples were derivatized using phenylisothio cyanate, dried, and metabolites were extracted using 40% methanol solution in water. All reagents were HPLC grade (Sigma Aldrich, Darmstadt, Germany).

The serum samples were thawed on ice, pipetted onto a 96-well plate (10 μL per sample) and derivatized using phenylisothiocyanate. A combination of flow injection analysis and liquid chromatography through a C18 column was used to determine the concentrations of metabolites.

## Figures and Tables

**Table 1:** Targeted analysis results from Mann–Whitney–Wilcoxon (MWW) test where AD patients’ lesional skin samples were compared to PS patients’ lesional skin samples.

Metabolite	AD-L Median Value	PS-L Median Value	AD-L vs. PS-L MWW *p*-Value FDR 5% Corrected	Median Fold Change (AD-L over PS-L)	Median Fold Change (PS-L over AD-L)
Biogenic amines					
ADMA	0.1622	0.4483	0.0043		2.8
Acylcarnitines					
C2	0.1504	0.2529	0.0086		1.7
C18.2	0.2241	0.4397	0.0318		2
Sphingolipids					
SM.C26.0	0.5119	0.2083	0.0028	2.5	
SM.C26.1	0.4966	0.2109	0.0035	2.4	
SM.OH.C22.1	0.6539	0.3365	0.0095	1.9	
SM.C18.1	0.5165	0.2443	0.0095	2.1	
SM.C24.1	0.6115	0.3218	0.0095	1.9	
SM.C24.0	0.6181	0.3434	0.0294	1.8	
SM.OH.C22.2	0.5429	0.3222	0.0328	1.7	
SM.C16.1	0.4554	0.3275	0.044	1.4	
SM.C16.0	0.5086	0.3374	0.044	1.5	
Glycerophospholipids					
PC.ae.C38.1	0.1106	0.241	0.0095		2.2
PC.ae.C36.0	0.0722	0.1618	0.0184		2.2
lysoPC.a.C16.1	0.1331	0.2108	0.0274		1.6
PC.ae.C38.0	0.1751	0.3319	0.0365		1.9
PC.ae.C38.2	0.1615	0.2585	0.044		1.6
PC.ae.C36.1	0.1518	0.2338	0.044		1.5
Metabolite ratios					
Orn…Arg	0.0063	0.0025	0.0285	2.5	

**Table 2:** Statistically significantly different metabolites between the blood serum samples from atopic dermatitis (AD) and psoriasis (PS) patients.

Metabolite	AD Serum Median Value	PS Serum Median Value	AD Serum vs. PS Serum MWW *p*-Value FDR 5% Corrected	Median Fold Change (PS Serum over AD Serum)
Amino acids				
Cit	0.1843	0.2806	0.0392	1.5
Glu	0.1146	0.2282	0.0392	2
Pro	0.1793	0.3073	0.0392	1.7
Acylcarnitines				
C0	0.154	0.4292	0.0184	2.8
C18.1	0.2312	0.3417	0.0392	1.5

**Table 3:** Clinical and demographic characteristics of the patients’ cohorts.

Patients’ Characteristics				
	Psoriasis (Skin)	Atopic Dermatitis (Skin)	Psoriasis (Serum)	Atopic Dermatitis (Serum)
Age, mean (y) (range)	46.5 (20–75)	32.1 (20–50)	46.3 (20–75)	33.4 (19–54)
Sex, no. (%)				
Male	13 (65)	4 (26.7)	37 (67.3)	6 (24)
Female	7 (35)	11 (73.3)	18 (32.7)	19 (76)
Ethnicity	Caucasian	Caucasian	Caucasian	Caucasian
PASI score, mean (range)	8.455 (2.8–22)		9.675 (1–34)	
Psoriasis, no. (%)				
Psoriatic associated joint involvement	5 (25)		15 (27.3)	
Psoriatic associated nail involvement	7 (35)		22 (40)	
Atopic dermatitis, no. (%)				
AD associated rhinitis		10 (66.7)		13 (52)
AD associated asthma		6 (40)		9 (36)
AD type, no. (%)				
normal IgE–intrinsic		2 (13.3)		5 (20)
elevated IgE–extrinsic		13 (86.7)		18 (72)
unknown				2 (8)
Family history				
positive	9 (45)	7 (46.7)	23 (41.8)	12 (48)
negative	10 (50)	8 (53.3)	24 (43.6)	10 (40)
unknown	1 (5)		8 (14.6)	3 (12)
Age of onset (%)				
≥40 years of age (PS)	7 (35)		12 (21.8)	
<18 years of age (AD)		14 (93.3)		23 (92)

## Data Availability

All data generated or analyzed during this study are included in this published article.
